# Role of Triple‐Negative Breast Cancer‐Derived Extracellular Vesicles in Metastasis: Implications for Therapeutics and Biomarker Development

**DOI:** 10.1111/jcmm.70448

**Published:** 2025-03-03

**Authors:** Xue Wan, Liqi Yang, Linjun Wu, Jiandong Lei, Jintao Li

**Affiliations:** ^1^ Department of Laboratory Medicine Leshan Hospital of Traditional Chinese Medicine Leshan China; ^2^ Department of Laboratory Medicine Leshan Maternal and Child Health Hospital Leshan China

**Keywords:** biomarker, cancer progression, extracellular vesicles, immune regulation, metastasis, resistance, therapeutics, triple negative breast cancer, tumour microenvironment

## Abstract

Triple‐negative breast cancer (TNBC) is a highly aggressive form of breast cancer with a poor prognosis and high mortality. The chemotherapeutic regimen remains the predominant treatment modality for TNBC in current clinical practice. However, chemotherapy resistance significantly complicates the development of an effective treatment regimen. Furthermore, the immunosuppressive microenvironment of TNBC contributes to enhanced tumour aggressiveness. Consequently, understanding its mechanisms of progression and finding effective therapeutic interventions is crucial. Recent evidence has identified extracellular vesicles (EVs) as key mediators of cell‐to‐cell communication in TNBC progression and immune regulation. In view of the remarkable ability of EVs to transfer active molecules, such as proteins and nucleic acids, from parental to recipient cells, they are regarded as a promising biomarker and novel drug delivery system. In this review, we provide an overview of how EVs derived from TNBC cells and tumour microenvironment cells play a role in regulating tumour progression. We also discuss the potential of EVs for immune regulation and their application as novel therapeutic strategies and tumour markers in TNBC. The knowledge gained from studying EV‐mediated communication in TNBC could lead to the development of targeted therapies and improve patient outcomes.

## 1 Introduction

Triple‐negative breast cancer (TNBC), which lacks oestrogen receptor (ER), progesterone receptor (PR), and human epidermal growth factor receptor 2 (HER2), accounts for approximately 20% of all breast cancers [1]. TNBC has a poor prognosis due to its rapid proliferation, early metastasis, and absence of therapeutic molecular targets. Recurrence and metastasis typically occur within 1–3 years, resulting in a lower 5‐year survival rate (76.9%, compared to 90.3% for other subtypes) [1, 2]. Cytotoxic chemotherapeutic agents, such as anthracyclines and taxanes, are effective against TNBC, but the development of drug resistance can lead to increased aggressiveness and metastasis of tumour cells [3]. Consequently, it is crucial to understand the molecular and cellular components involved in the pathogenesis and progression of tumour in order to develop new strategies for preventing and controlling this process.

**FIGURE 1:**
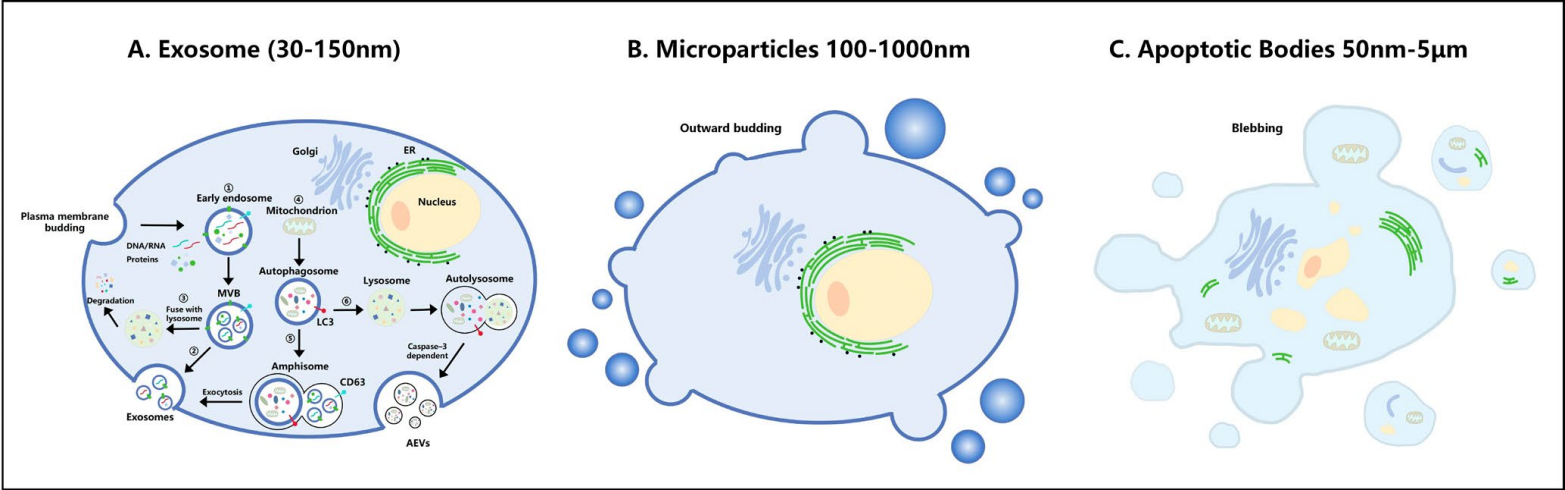
The composition and biogenesis of EV. (A) The biogenesis of exosomes. ①, ② MVBs are derived from endocytic mechanisms. Distinct mechanisms mediate plasma membrane budding and early endosome formation, further facilitating their maturation into MVBs, which fuse with the plasma membrane to release exosomes. ③ MVBs fuse with lysosomes to be degraded. ④, ⑤ LC3‐positive autophagosomes fuse with CD63‐positive MVBs to generate amphisomes, which are subsequently released as exosomes outside the cells through exocytosis. ⑥ Autophagosomes merge with lysosomes to form autolysosomes and release apoptotic exosome‐like vesicles (AEVs) into the extracellular space in a caspase‐3‐dependent manner. (B) MPs are released through budding from the plasma membrane. (C) Apoptotic bodies are released through blebbing by apoptotic cells.

Initially, extracellular vesicles (EVs) were considered platelet‐derived pro‐coagulant particles in plasma. Accumulating research evidence has highlighted their significance as a crucial means of intercellular communication through autocrine, paracrine, or endocrine mechanisms [4]. EVs are membrane‐bound structures that are actively secreted by almost all types of cells and released into the extracellular space. EVs specifically target cells by delivering various cargo, including nucleic acids, proteins, metabolites, and other bioactive molecules to modulate their functional characteristics [5]. Additionally, EVs are widely present in bodily fluids, including blood, urine, cerebrospinal fluid, and saliva. Their lipid bilayer structure protects their contents from degradation, making EVs a potential source of disease biomarkers [1]. These vesicles can be classified into two size groups: small EVs (sEVs) with a diameter less than 200 nm and medium/large EVs with a diameter greater than 200 nm [6]. They are further subdivided based on their biogenesis pathways into exosomes (30–150 nm), microparticles (100–1000 nm), and apoptotic bodies (50 nm–5 μm) [7, 8, 9]. Exosomes are vesicles formed through the fusion and secretion of multivesicular bodies (MVBs) with the plasma membrane [5]. The MVBs function as the central process in exosome biogenesis, encompassing membrane budding and generation of intraluminal vesicles (ILVs). These activities involve both endosomal sorting complex required for transport (ESCRT)‐dependent and ESCRT‐independent pathways [4, 10]. The ESCRT‐dependent pathway relies on four core ESCRT complexes (ESCRT‐0, I, II, and III), which collaboratively facilitate the stepwise formation of ILVs. The ESCRT‐independent pathway depends on intricate lipids and other protein‐related pathways, such as ceramide, Rabs, and tetraspanins (CD63) [10, 11, 12]. Furthermore, the autophagosome is intricately associated with exosomal secretion. Two outcomes ensue: (1) the formation of amphisomes facilitates exosome secretion; (2) it can release apoptotic exosome‐like vesicles (AEVs) in a caspase‐3‐dependent manner [4, 5] (Figure 1A). Microparticles (MPs) or microvesicles (MVs) are formed by the outward budding and fission of the cellular membrane and subsequently released into the extracellular milieu during cellular activation, injury, or apoptosis across diverse cell types [13] (Figure 1B). Apoptotic bodies are a distinct subtype of EVs, which are formed through plasma membrane blebbing and apoptotic membrane protrusion during the process of apoptosis [14, 15] (Figure 1C).

**FIGURE 2:**
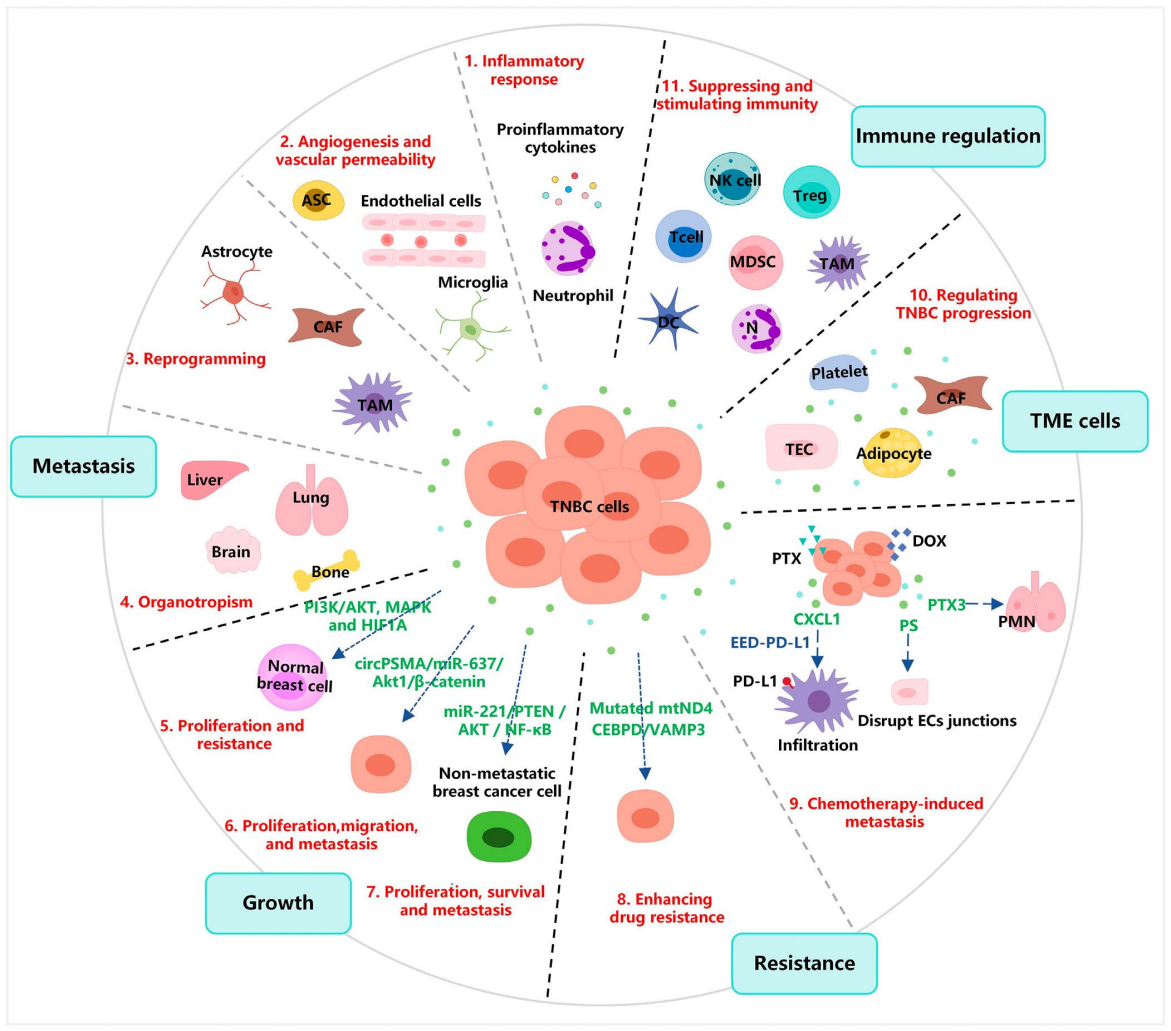
The pro‐tumour effects and immune regulation of TNBC‐EVs. Metastasis: (1)–(4) display that TNBC‐EVs facilitate PMN establishment through inflammatory response, angiogenesis, and vascular permeability, reprogramming, and organotropism to drive distant tumour dissemination. Growth: (5) TNBC‐EVs induce proliferation and drug resistance in normal breast cells. (6) TNBC‐EVs often trigger the proliferation, migration, and metastasis of TNBC in an autocrine manner. (7) TNBC‐EVs also promote proliferation, survival, and metastasis of non‐metastatic breast cancer cells. Resistance: (8) TNBC‐EVs enhance drug resistance of TNBC cells. (9) Drug‐resistant TNBC cells also secrete EVs to facilitate TNBC metastasis. TME cells: (10) TME cell‐EVs can regulate TNBC progress, including CAFs, platelets, TECs, and adipocytes. Immune regulation: (11) EVs in TNBC involve a diverse array of immune regulatory mechanisms, encompassing T cells, NK cells, DCs, MDSCs, TAMs, Tregs, and neutrophils.

The high invasiveness, heterogeneity, and immunosuppressive tumour microenvironment (TME) of TNBC might be primary factors contributing to its early metastasis, recurrence, and drug resistance [16]. Since EVs could transfer the phenotypic characteristics representing their origin cells to recipient cells [17], they play an important role in these processes, as progressively demonstrated in the literature. It has been shown that TNBC‐derived EVs containing miR‐125b cause fibroblasts (NFs) to differentiate into cancer‐associated fibroblasts (CAFs), resulting in tumour growth and metastasis [18]. Exosomal integrin α_6_β_4_ and α_v_β_5_ produced by TNBC cells could contribute to the establishment of the pre‐metastatic niche (PMN), which, in turn, facilitates distant organ metastasis [19]. Another study has revealed that exosomal miR‐423‐5p derived from cisplatin‐resistant MDA‐MB‐231 cells enhances the resistance of sensitive breast cancer cells to cisplatin [20]. EVs have distinct inherent transport capability, tumour tropism, and modifiability, and are proposed as promising drug delivery carriers. A novel therapy based on natural or synthetic EVs is rapidly evolving into significant and successful treatment regimens for TNBC. Besides, the circulation, stability, and specificity of EVs in body fluids might provide reliable biomarkers for the diagnosis, prognosis, and chemotherapy efficacy evaluation of TNBC. Herein, this review aims to summarise the current understanding of how EVs regulate pro‐tumour effects in TNBC, while also discussing the potential of EVs for immune regulation and their application as novel therapeutic strategies and tumour markers in TNBC. Figure 2 briefly illustrates how EVs involve metastasis, growth, chemoresistance, and immune regulation of TNBC in different ways.

## 2 The Role of Tumour‐Derived EVs in the Progression of TNBC

Intercellular communication, a crucial aspect of cancer progression and metastasis, induces alterations in the microenvironment and impacts tumour growth and dissemination. Numerous studies have substantiated the active involvement of EVs in the progression of TNBC, primarily encompassing tumour metastasis and growth.

### 2.1 EVs In TNBC Metastasis

#### 2.1.1 EVs Inducing Pre‐Metastatic Niche Formation

The entry of circulating tumour cells (CTCs) into secondary or distant organ sites is a critical step in tumour metastasis. However, the local microenvironment that CTCs encounter plays a pivotal role in determining whether tumour cell colonisation can occur [21]. Over the years, numerous studies have demonstrated that primary tumour cells initiate the formation of a supportive microenvironment in secondary organ or tissue sites conducive to tumour metastasis, known as the PMN. The PMN exhibits various tumour‐associated properties, including inflammation, angiogenesis and vascular permeability, lymphangiogenesis, reprogramming, immunosuppression, and organotropism [21]. Accumulating evidence suggests that PMN formation is closely associated with soluble factors secreted by primary tumour cells into the systemic circulation, particularly tumour‐derived EVs [22]. Table 1 briefly summarises the role of tumour‐secreted EVs in PMN formation.

**TABLE 1:** The role of tumour‐secreted EVs in PMN formation.

Type	Contents in EVs	Type of EVs	Specific mechanism	Target organ	Ref. No.
Inflammation	No specific	EV	Release of NETs, ROS, IL8, VEGF, and MMP‐9 to promote the transition of neutrophils into a N2‐like phenotype	No specific	[23]
	CEMIP	Exosome	Inducing a proinflammatory vascular niche by upregulating of cytokines *Ptgs2*, *Tnf*, and *Ccl*/*Cxcl*	Brain	[24]
Angiogenesis	Diacylglycerol	EV	Activating the PKD signalling pathways in endothelial cells	No specific	[25]
	miR‐182‐5p	EV	Enhancing endothelial cells proliferation, migration, and angiogenesis through regulating the CMTM7/EGFR/AKT axis	Lung	[26]
	CEMIP	Exosome	Upregulating of cytokines *Ptgs2*, *Tnf*, and *Ccl*/*Cxcl*	Brain	[24]
Vascular permeability	miR‐939	Exosome	Downregulation of VE‐cadherin expression and disruption of barrier integrity in endothelial cells	No specific	[27]
	miR‐105	Exosome	Heightening vascular permeability within the pulmonary microenvironmen by suppressing the expression of ZO‐1	Lung	[28]
	NDPK‐B phosphotransferase activity	EV	Activating the expression of P2Y1 purinergic receptors on vascular endothelial cells by maintaining extracellular ATP and ADP pools	Lung	[29]
Reprogramming	No specific	EV	Activating α‐SMA^+^ myCAF subsets	Lung	[30]
	MALAT1	EV	Inducing macrophages M2 polarisation via the POSTN/Hippo/YAP axis	No specific	[31]
	No specific	EV	CCL5 regulating EVs educate macrophages towards TAMs	Lung	[32]
	ITGB4	Exosome	Triggering glycolysis in CAFs through BNIP3L‐dependent mitophagy mechanism	No specific	[33]
	CircSIPA1L3	Exosome	Increasing mRNA stability of SLC16A1 and RAB11A through either USP7‐mediated deubiquitination of IGF2BP3 or sponging miR‐665 to enhance glycolytic metabolism	No specific	[34]
	miRNA‐122	EV	Suppressing glucose uptake through downregulating the glycolytic enzyme PKM in PMN cells	Brain Lung	[35]
	miR‐199b‐5p	EV	Impairing the metabolic coupling between neurons and astrocytes via targeting solute carrier transporters	Brain	[36]
Organotropism	No specific	EV	Changing the extracellular matrix and soluble components of the lung microenvironment	Lung	[37]
	LAP‐TGFβ1	Exosome	Remodelling the pulmonary vascular niche by reducing ZO‐1 expression	Lung	[38]
	No specific	EV	CX3CL1–CX3CR1 signalling in the liver PMN upregulated MMP9, promoting macrophages migration and cancer cells invasion	Liver	[39]
	TGFβ1	EV	Enhancing tumour cells adhesion to the liver microenvironment by upregulating fibronectin in liver sinusoidal endothelial cells	Liver	[40]
	PDGF‐BB, CCL3, CCL27, VEGF, Angiopoietin 2	EV	Stimulating the production of RANKL‐positive EVs from osteoblasts and promoting osteoclastogenesis and angiogenesis	Bone	[41]
	miR‐218	EV	Downregulating *COL1A1* expression in osteoblasts to regulate collagen deposition and enhance osteolysis	Bone	[42]
	miRNA‐181c	EV	Disrupting the blood–brain barrier integrity through involving the degradation of PDPK1 and activating cofilin‐induced modulation of Actin dynamics	Brain	[43]

#### 2.1.2 Tumour‐derived EVs and Inflammatory Molecules

It is well‐established that chronic inflammation is a risk factor for the development of cancer. In this context, cytokines play a significant role in promoting abnormal inflammatory signalling pathways and recruiting immunosuppressive cells to trigger tumour metastasis [44]. A study has demonstrated that MDA‐MB‐231 cells release EVs that create a chemoattractant environment for neutrophils. These EVs promote the transition of neutrophils into an N2 phenotype marked by increased release of neutrophil extracellular traps (NETs), production of reactive oxygen species (ROS), and stimulation of the secretion of IL8, VEGF, and MMP9, which enhance the viability of tumour cells [23]. It was also found that exosomes derived from brain metastatic MDA‐MB‐231 cells containing cell migration‐inducing and hyaluronan‐binding protein (CEMIP) facilitated brain metastasis by inducing a proinflammatory vascular niche through the upregulation of cytokines *Ptgs2*, *Tnf*, and *Ccl*/*Cxcl* [24]. Therefore, EVs play a critical role in promoting the establishment of an inflammatory environment for tumour metastasis by upregulating proinflammatory cytokines and activating inflammation.

#### 2.1.3 Tumour‐derived EVs and Angiogenesis and Vascular Permeability

Angiogenesis and increased vascular permeability play vital roles in the promotion of PMN formation. Angiogenic factors provide the necessary nutrients for the rapid growth of PMN and the subsequent metastasis of cancer cells. TNBC‐derived EVs are known for their ability to induce angiogenesis, leading to distant organ metastasis. According to research by Nishida‐Aoki et al. [25], EVs from highly metastatic TNBC cells accumulated diacylglycerols. These diacylglycerols stimulated tumour angiogenesis and progression by activating the protein kinase D (PKD) signalling pathways in endothelial cells. It has been demonstrated that miR‐182‐5p, enclosed within EVs secreted by MDA‐MB‐231 cells, can be taken up by endothelial cells. This uptake enhances endothelial cell proliferation, migration, and angiogenesis to promote lung metastasis through regulating the CKLF‐like MARVEL transmembrane domain‐containing 7(CMTM7)/EGFR/AKT signalling axis [26]. Moreover, the internalisation of CEMIP^+^ exosomes, derived from MDA‐MB‐231 cells with a propensity for brain metastasis, by brain endothelial and microglial cells initiates an inflammatory pathway. This leads to the upregulation of genes such as *Ptgs2*, *Tnf*, and *Ccl*/*Cxcl*, promoting vascular remodelling and angiogenesis [24].

Loss of endothelial cell membrane adhesion molecules like VE‐cadherin and tight junction 1 (ZO‐1) has been identified as a crucial step in tumour development and metastasis. The disruption of the vascular barrier and increased vascular permeability are associated with this process [27]. In the case of TNBC cells, exosomal miR‐939 specifically targets human vascular endothelial cells, resulting in the downregulation of VE‐cadherin expression and the disruption of barrier integrity [27]. In a study by Zhou et al. [28], it was discovered that highly metastatic TNBC cells secreted exosomal miR‐105, which was internalised by pulmonary microvascular endothelial cells and heightened vascular permeability within the pulmonary microenvironment by suppressing the expression of ZO‐1 in these cells. Consequently, this facilitated the subsequent colonisation of tumour cells in the lung. Besides, Duan et al. [29] demonstrated that MDA‐MB‐231 EVs exhibited an abundance of nucleoside diphosphate kinase B (NDPK‐B) phosphotransferase activity. These EVs activated the expression of P2Y1 purinergic receptors on vascular endothelial cells by maintaining extracellular ATP and ADP pools to increase vascular permeability and promote the formation of lung PMN.

#### 2.1.4 Tumour‐Derived EVs and Reprogramming

Tumour cells employ EVs to recruit and reprogram non‐malignant stromal cells into PMN to facilitate tumour metastasis [21]. The tumour stroma predominantly consists of CAFs that display heterogeneity and phenotypic plasticity. In a study by González‐Callejo et al. [30], EVs derived from TNBC stem cells were found to activate α‐SMA^+^ myofibroblast (myCAFs) subsets and establish a PMN supportive of tumour cell proliferation in the lung. Tumour‐associated macrophages (TAMs) display various phenotypes upon exposure to different stimuli; M1 polarisation is associated with a pro‐inflammatory response, while M2 polarisation is linked to an anti‐inflammatory effect. During tumour progression, TAMs undergo remodelling processes that resemble M2 polarisation [45]. TNBC‐EV‐derived lncRNA, metastasis‐associated lung adenocarcinoma transcript 1 (MALAT1), can induce macrophages M2 polarisation and facilitate the occurrence and metastasis of TNBC via the POSTN/Hippo/YAP axis [31]. C‐C Motif Chemokine Ligand 5 (CCL5) expression in TNBC cells has been shown to regulate EVs biogenesis/secretion/cargo, as well as educate macrophages through EVs towards TAMs with a pro‐metastatic phenotype and increase tumour metastasis to the lung [32].

Due to nutrient deficiencies in the TME, tumour cells undergo metabolic reprogramming to sustain rapid proliferation. This adaptation is mainly facilitated by microenvironmental cells, which provide nourishment for tumour cells and regulate tumour metabolism to remodel PMN [30, 33]. When TNBC cells overexpressing the integrin β4 (ITGB4) were co‐cultured with CAFs, it was observed that the TNBC cells transferred ITGB4 to CAFs via exosomes. This transfer triggered glycolysis in CAFs through a BCL2 interacting protein (BNIP3L)‐dependent mitophagy mechanism, ultimately promoting the proliferation, epithelial –mesenchymal transition (EMT), and invasion of TNBC cells [33]. Exosomal circSIPA1L3 increased mRNA stability of the lactate export carrier SLC16A1 and the glucose intake enhancer RAB11A through either USP7‐mediated deubiquitination of IGF2BP3 or sponging miR‐665, leading to enhanced glycolytic metabolism in TNBC [34]. In addition, Fong et al. [35] reported that MDA‐MB‐231 cells upregulated the secretion of miRNA‐122 in EVs. This upregulation promoted distant organ metastasis (brain and lung) by suppressing glucose uptake through downregulating the glycolytic enzyme pyruvate kinase (PKM) in PMN cells (lung fibroblasts, astrocytes). EV miR‐199b‐5p secreted by brain‐tropic MDA‐MB‐231 cells impairs the metabolic coupling between neurons and astrocytes to facilitate the development of brain metastasis via targeting solute carrier transporters (SLC1A2/EAAT2 in astrocytes and SLC38A2/SNAT2 and SLC16A7/MCT2 in neurons) [36].

In summary, EV‐mediated intercellular communication plays a crucial role in regulating the reprogramming of microenvironment cells and actively contributes to tumour progression and metastasis. Additionally, certain stromal cells are also implicated in reprogramming to support tumorigenic properties. However, their specific involvement in reprogramming in TNBC remains unexplored.

#### 2.1.5 Tumour‐Derived EVs and Organotropism

Organotropism refers to the preference of certain types of tumours to metastasize to specific organs. In breast cancer, cavitary epithelial breast cancers primarily metastasize to the bones, while TNBC has a tendency to spread to visceral organs. Recent studies have highlighted the role of EVs in mediating organ‐specific metastasis. Before the initiation of metastasis, primary tumours release EVs into the bloodstream, acting as messengers to facilitate the subsequent dissemination and colonisation of specific target organs by tumour cells [46].

In the case of lung metastasis, TNBC changes the extracellular matrix and soluble components of the lung microenvironment using tumour‐derived EVs, thereby promoting PMN establishment [37]. LAP‐TGFβ1 is a protein dimer composed of latency‐associated peptide (LAP) and the TGFβ1 active subunit. Acetylated, this protein is transported into exosomes, which can remarkably remodel the pulmonary vascular niche by reducing ZO‐1 expression in lung endothelial cells [38]. In liver metastasis, TNBC cell‐derived EVs also promoted the liver macrophages to secrete tumour necrosis factor‐alpha (TNFα) and induced the liver endothelial cells to upregulate the CX3CL1. Increased expression of CX3CL1 recruits the CX3CR1‐positive macrophages to liver PMN, resulting in the upregulation of MMP9, which facilitated the migration of immune cells and cancer cells [39]. Kim et al. [40] discovered that EVs isolated from TNBC patients with liver metastasis exhibited elevated levels of TGFβ1, which enhanced tumour cell adhesion to the liver microenvironment by upregulating fibronectin in liver sinusoidal endothelial cells. In the context of bone metastasis, osteotropic MDA‐MB‐231 cells released EVs containing various molecular mediators such as Platelet‐Derived Growth Factor (PDGF)‐BB, CCL3, CCL27, VEGF, and Angiopoietin 2. These EVs stimulated the production of receptor activators of nuclear factor kappa‐B ligand (RANKL)‐positive EVs from osteoblasts and promoted osteoclastogenesis and angiogenesis [41]. Bone‐tropic MDA‐MB‐231 cell‐derived EV miR‐218 directly attenuated collagen type I alpha 1 chain (*COL1A1*) expression in osteoblasts, consequently compromising collagen deposition and enhancing osteolysis [42]. Finally, in the case of brain metastasis, EVs derived from brain‐metastatic TNBC cells containing miRNA‐181c disrupted the blood–brain barrier integrity by involving the degradation of 3‐phosphoinositide‐dependent protein kinase‐1 (PDPK1) and activating cofilin‐induced modulation of actin dynamics [43].

In conclusion, EVs derived from TNBC cells undergo significant reprogramming of the microenvironment in target organs, creating a more favourable milieu for tumour growth and implantation during organotropic metastasis. The investigation of EVs' affinity towards specific organs also provides valuable insights and methodologies to combat distant organ metastasis in TNBC.

### 2.2 EVs In TNBC Growth

In TNBC growth, it has been observed that HCC1806 TNBC cells secrete EVs to facilitate proliferation and confer drug resistance in MCF10A cells through PI3K/AKT, MAPK, and HIF1A signalling pathways [47]. The exosomes from TNBC cells exhibited a high abundance of circPSMA, which facilitated growth, migration, and metastasis through the miR‐637/Akt1/β‐catenin (cyclin D1) axis [48]. Furthermore, certain EV miRNAs have been implicated in tumour growth, exemplified by the transfer of miR‐221 from TNBC‐derived microvesicles to non‐metastatic breast cancer cells, thereby enhancing receptor cell proliferation, survival, and metastasis by inducing EMT via activating AKT Ser/Thr kinase/NF‐κB through targeting phosphatase and tensin homologue (PTEN) [49].

## 3 EVs In TNBC Resistance

Chemotherapy remains the primary therapeutic modality for TNBC. However, only around 30% of TNBC patients show a complete response to this treatment, and some even experience chemotherapy‐induced metastasis [50]. Tumour cells evolve chemoresistance via multiple mechanisms, including drug efflux, drug metabolism, epigenetic modification, target mutation, and alteration of signalling pathways [51, 52]. EVs that transport bioactive molecules play a crucial role in conferring chemoresistance to tumour cells, which has emerged as a significant mechanism underlying tumour drug resistance, such as acquired drug resistance and gene transcriptional regulation. The EVs of chemotherapy‐resistant TNBC cells could transfer mitochondria to sensitive cancer cells, thereby augmenting their resistance. These exosome fractions originating from mitochondria‐resistant TNBC cells contributed to the development of acquired resistance by elevating mtDNA with mutated mtND4 gene [53]. CCAAT enhancer binding protein delta (CEBPD) and vesicle‐associated membrane protein 3 (VAMP3) exhibited up‐regulation in chemotherapy‐resistant tissues as well as PTX‐resistant TNBC cells. Notably, CEBPD promoted autophagy activation by transcriptionally activating VAMP3, thereby augmenting the chemotherapy resistance of TNBC [54].

Furthermore, clinical studies now offer substantial evidence that the cytotoxic effects of chemotherapy are linked to the formation of a metastatic microenvironment. One of the mechanistic principles involved is the chemotherapy‐induced release of metastatic EVs from tumour cells [51]. The use of doxorubicin (DOX) has been found to increase the secretion of sEVs by TNBC cells. Proteomic analysis of these sEVs has revealed an enrichment of the inflammatory glycoprotein pentraxin 3 (PTX3), which primes the PMN and accelerates lung metastasis [50]. DOX also led to the release of phosphatidylserine‐positive microparticles (PS^+^ MPs) from TNBC cells. These MPs initiated a local coagulation cascade and disrupted the junctions in endothelial cells, ultimately facilitating transendothelial migration of cancer cells [55]. Additionally, paclitaxel (PTX) induced apoptosis of TNBC cells to release CXCL1‐enriched EVs, which were efficiently phagocytosed by macrophages. This process enhanced the infiltration of immunosuppressive programmed cell death 1 ligand 1 (PD‐L1)‐expressing TAMs to promote TNBC growth and lung metastasis through transcriptionally activating Embryonic ectoderm development protein (EED)‐mediated PD‐L1 promoter activity [56].

Given the critical role of EVs in tumour drug resistance, they are now regarded as promising therapeutic targets. This offers a novel perspective for developing treatment strategies for TNBC by identifying or inhibiting the drug resistance signals carried by EVs.

## 4 Functions of Tumour Microenvironment Cell‐Derived EVs in TNBC Progression

**FIGURE 3:**
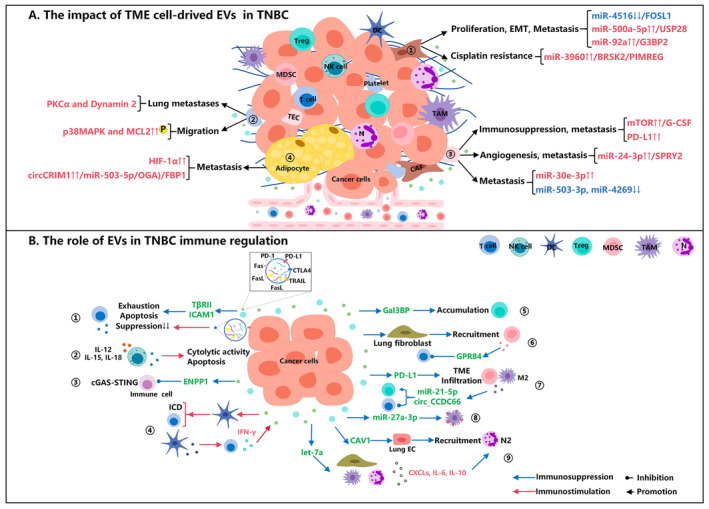
(A) The impact of tumour microenvironment cell‐derived EVs in TNBC. ① CAFs secrete EVs that are internalised by cancer cells to induce proliferation, EMT, metastasis, and drug resistance. ② PEVs accelerate cancer cells metastasis.③ EVs secreted by TECs facilitate immunosuppression, angiogenesis, and metastasis in TNBC. ④ Adipocyte‐derived EVs enhance tumour metastasis. (B) The role of EVs in TNBC immune regulation. ① Cancer cell‐derived EVs induce T cell exhaustion and apoptosis, while T cell‐derived EVs can reduce immune suppression. ② EVs secreted by NK cells can induce cytolytic activity and apoptosis in cancer cells. ③ Tumour cell‐derived EVs can hinder cGAS‐STING signalling in immune cells. ④ Cancer cell‐derived EVs induce ICD of DCs and promote Th1 cell differentiation, and DC‐derived EVs can augment the activation of T cells to secrete IFN‐γ. ⑤ Cancer cell‐derived EVs result in an increase in Tregs in tumours. ⑥ Cancer cell‐derived EVs can contribute to MDSC recruitment by increasing the expression of chemokines CXCL1 and CXCL8 in lung fibroblasts. MDSCs producing EVs can induce T cell senescence. ⑦ Cancer cell‐derived EVs can promote the infiltration of M2 TAMs and MDSCs in the TME. TAMs secreting EVs can lead to a decrease in T cells and an increase in Tregs within the TME. ⑧ Cancer cell‐derived EVs can upregulate PD‐L1 expression in macrophages. ⑨ Cancer cell‐derived EVs can enhance recruitment and N2 polarisation of neutrophils by inducing the production of CXCLs, IL‐6, and IL‐10.

The TME is an internal milieu characterised by hypoxia, low pH, and nutrient deprivation that tumour cells depend on for survival and development. It consists primarily of immune cells, fibroblasts, platelets, endothelial cells, adipocytes, and various other tissue‐resident cells. The interaction between tumour cells and the TME plays a crucial role in driving tumour progression, wherein EVs secreted by TME cells can directly influence the behaviour of tumour cells to intricately regulate various facets of tumour progression (Figure 3A).

### 4.1 Cancer‐Associated Fibroblasts

CAFs are a significant component of the TME in most solid malignancies [57]. Compared to NFs, the expression of miR‐4516 was downregulated in CAFs; CAF‐EVs generally enhanced the proliferation of TNBC cells by upregulating the expression of FOSL1, a target gene of miR‐4516 [58]. Through the miR‐500a‐5p/ubiquitin specific peptidase 28 (USP28) axis, exosomal miR‐500a‐5p in CAFs could promote cancer cell proliferation, EMT, and metastasis [57]. Moreover, RNA‐binding protein SNRPA also facilitated the transfer of CAF‐derived exosomal miR‐92a to breast cancer cells, which promoted cancer cell invasion and metastasis by downregulating G3BP2 [59]. Additionally, CAFs can involve drug resistance in TNBC. Superoxide dismutase 1(SOD1)‐high fibroblasts transmit exosomal miR‐3960 to facilitate cisplatin resistance in TNBC by inhibiting PIMREG phosphorylation mediated by BRSK2, a member of the AMPK protein kinase family [60].

### 4.2 Platelets

Platelet‐derived extracellular vesicles (PEVs) have emerged as important regulators of platelet‐mediated metastasis and cancer progression. Higher levels of PEVs in cancer patients are associated with an unfavourable prognosis [61]. Zhao et al. [62] demonstrated that activated protein kinase Cα (PKCα) cooperated with Dynamin 2 to induce PEV production and facilitated lung metastases in breast cancer. When metastatic MDA‐MB‐231 cells internalised PEVs, they triggered a sustained increase in intracellular calcium levels and stimulated the phosphorylation of migration‐related proteins such as p38MAPK and myosin light chain 2 (MCL2) [61].

### 4.3 Tumour‐Associated Endothelial Cells

Tumour‐associated endothelial cells (TECs) provide essential nutrients and oxygen, immune cells, and facilitate the dissemination of tumour cells to secondary sites [63]. A study by Koni et al. [63] showed that EVs enriched with mTOR released by TECs induced the secretion of granulocyte‐colony stimulating factor (G‐CSF) from myeloid cells and cancer cells. This led to an increase in myeloid‐derived suppressor cells (MDSCs) and T cell exhaustion, supporting tumour immunosuppression and metastatic growth. The interleukin (IL)‐3/IL‐3Rα signalling in TECs modulated the release of pro‐metastatic EVs, which exerted immunosuppressive effects by upregulating PD‐L1 expression in peripheral blood mononuclear cells (PBMCs) and TNBC cells. Conversely, blocking IL‐3Rα on TECs promoted the secretion of miR‐214‐enriched EVs, thereby restoring aberrant anti‐tumour immune responses [64]. Furthermore, TECs generated EVs by blocking IL‐3Rα or depleting miR‐24‐3p, which could also effectively inhibit angiogenesis and impede primary tumour metastasis by upregulating sprouty RTK signalling antagonist 2 (SPRY2) in TNBC [65]. ELK3, an ETS domain‐containing protein expressed in lymphatic endothelial cells (LECs), promotes the dissemination of MDA‐MB‐231 cells through exosomal oncogenic miRNA (miR‐30e‐3p) and oncosuppressive miRNAs (miR‐503‐3p and miR‐4269) [66].

### 4.4 Adipocytes

Obesity is a significant risk factor for TNBC. Adipocyte‐derived EVs induced the activity of hypoxia‐inducible factor‐1α (HIF‐1α), thus stimulating the metastasis of TNBC cells [67]. Adipocyte‐derived exosomal circCRIM1 enhanced TNBC evolution and metastasis by inhibiting miR‐503‐5p and activating the O‐GlcNAcase (OGA)/Fructose‐Bisphosphatase 1 (FBP1) pathway [68].

## 5 Emerging Role of EVs in TNBC Immune Regulation

Within the TME, tumour cells frequently engage in communication with immune cells to facilitate tumour immunosuppression and evade immune surveillance to support tumour progression [69]. Tumour cells frequently drive tumour progression by impairing the anti‐tumour functionality of immune cells, such as T cells, natural killer (NK) cells, and dendritic cells (DCs), while also fostering the accumulation and activation of immunosuppressive cell populations, including regulatory T cells (Tregs), TAMs, tumour‐associated neutrophils, and MDSCs [69, 70, 71, 72]. Numerous studies have revealed that primary tumour cells release factors that trigger the mobilisation of immune cells, with EVs playing a crucial role in this process (Figure 3B).

### 5.1 T Cells

The impaired function or exhaustion of T cells is widely recognised as a characteristic feature of tumour‐induced immunosuppression. TNBC cell‐derived exosomes carrying surface PD‐L1, PD‐1, Fas, FasL, TNF‐related apoptosis‐inducing ligand (TRAIL), cytotoxic T lymphocyte antigen 4 (CTLA4), and TGFβ1 induced apoptosis in CD8^+^ T and CD4^+^ T cells. The entry of exosomes into T cells initiated apoptosis through the release of cytochrome C and second mitochondria‐derived activator of caspases (Smac) from mitochondria and cleavage of caspase‐3 and PARP in the cytosol [73]. In another study, breast cancer cell –EVs transfer the active TGFβ type II receptor (TβRII) and stimulate TGFβ signalling in CD8^+^ T cells. This induces the activation of SMAD3, which partners with T cell factor‐1 (TCF1) to impose CD8^+^ T cell exhaustion [74]. Similarly, TNBC cells secreting exosomal ICAM1 initiate the establishment of an immunosuppressive microenvironment that conduces to TNBC tumour growth and bone metastasis by mediating the depletion of CD8^+^ T cells [75]. Tregs are a subgroup of T cells with immunosuppressive effect. Raiter et al. [76] identified Galectin 3 binding protein (Gal3BP) in sEVs derived from TNBC patients' plasma. This protein and TNBC‐secreted Galectin 3 form a Gal3BP/Gal3 complex that induces an increase in Tregs and the release of immunosuppressive cytokines IL‐10 and IL‐35 via the CD45 signalling pathway.

EVs derived from T cells carrying cargoes have been identified as a pivotal factor for anti‐tumour responses. Activated T cells releasing exosomal PD‐1 interacted with PD‐L1‐containing TNBC cells or exosomes, thereby disrupting the direct interaction between T cells and TNBC cells, ultimately mitigating the suppressive effect of TNBC cells on activated T cells triggered by PD‐L1 [77].

### 5.2 Natural Killer Cells

NK cells are an integral component of the innate immune system, exerting direct cytotoxic effects or producing cytokines such as IFN‐γ and TNFα, which play crucial roles in anti‐tumour responses and immunomodulation [78]. NK cell‐EVs enhanced cytolytic activity against human cancer cell lines (glioblastoma, breast cancer, and thyroid cancer) and increased the expression of molecules associated with NK cell cytotoxicity by priming with IL‐15 [79]. IL‐12, IL‐15, and IL‐18 cultured human memory‐like NK cells secreting sEV entered tumour cells via macropinocytosis, thereby inducing cell death by activating the caspase‐dependent apoptotic pathway [80].

### 5.3 Dendritic Cells

DCs are specialised antigen‐presenting cells that elicit both innate and adaptive immunity in vivo. They release EVs that possess the ability to imitate the functions of DCs, exhibiting superior efficacy and safety in breast cancer treatment compared to DCs [81]. The cGAS‐STING pathway is a crucial innate immune response pathway. A finding reveals that tumour exosomal ENPP1 hinders cGAS‐STING signalling by hydrolysis of 2′3′‐cyclic GMP‐AMP (cGAMP) in immune cells, thereby facilitating tumour immune evasion [82]. TNBC‐derived exosomes can induce immunogenic cell death (ICD) and promote Th1 cell differentiation by enhancing the maturation and functionality of DCs [83].

Additionally, uptake of DC‐derived (EVs) by breast cancer cells can augment the activation of tumour‐sensitised T cells to secrete IFN‐γ for promoting a more potent tumour‐specific immune response [84].

### 5.4 Myeloid‐Derived Suppressor Cells

MDSCs represent a heterogeneous population of myeloid cells derived from immature myeloid precursors, exhibiting potent immunosuppressive capability. Their proliferation within TME is closely linked to the process of tumour colonisation and growth [85]. The activation of S100A10 in lung fibroblasts via tumour‐derived exosomes can increase the expression of chemokines CXCL1 and CXCL8, contributing to MDSCs recruitment and lung PMN formation [85].

Furthermore, MDSCs can transfer G‐protein‐coupled receptor 84 (GPR84) to CD8^+^ T cells by secreting exosomes and induce T cell senescence via the p53 signalling pathway, ultimately inhibiting T cell proliferation and function [86].

### 5.5 Tumour‐Associated Macrophages

In TAMs, TNBC cells released MPs loaded with PD‐L1, which mediated the differentiation of macrophages into an immune‐suppressive M2 phenotype to suppress the immune microenvironment through the activation of the TANK‐binding kinase 1 (TBK1)/signal transducer and activator of transcription 6 (STAT6) pathway and suppression of the AKT/mTOR pathways [87]. Similarly, exposure to oscillatory strain enhanced the production of PD‐L1^+^ exosomes by TNBC cells. In parallel, MDSCs and M2 macrophages facilitated their own infiltration by internalising these exosomes, thereby modulating the immunosuppressive characteristics of the TME [88]. Additionally, endoplasmic reticulum stress‐induced MD‐MB‐231 cells producing exosomal miR‐27a‐3p upregulated PD‐L1 expression in macrophages by activating the PTEN‐AKT/PI3K axis, finally promoting immune evasion [89].

TAM‐derived EVs are also implicated in the modulation of immune responses. EVs derived from M2 macrophages secreted miR‐21‐5p [90] and circCCDC66 [91], both of which lead to a decrease in T cells and an increase in Tregs within TME.

### 5.6 Tumour‐Associated Neutrophils

The presence of neutrophils in tumours is characterised by two phenotypes: N1 (anti‐tumour) or N2 (pro‐tumour). The upregulation of integrin α6β4 facilitated the internalisation of sEVs caveolin‐1 (CAV1) from breast cancer cells by lung epithelial cells, which enhanced neutrophil recruitment and N2 polarisation in pulmonary PMN through Toll‐like receptor 4 (TLR4) signalling [92]. Moreover, Qi et al. [71] found low‐let‐7 s exosomes from Lin28B‐induced breast cancer stem cells resulted in increased production of CXCLs, IL‐6, and IL‐10 by lung fibroblasts, neutrophils, and macrophages. This led to recruitment and N2 conversion of neutrophils and the formation of an immune‐suppressive lung PMN.

Apparently, these EVs play a dual role in tumour immunity by inducing both inhibitory and effector immune responses. A more comprehensive understanding of the distinctive functional capabilities of EVs in anti‐tumour immunity could facilitate the development of more efficacious anti‐tumour immunotherapies.

## 6 The Role of EVs in Immunotherapeutic Potential in TNBC

The lack of specific targets for TNBC poses a significant challenge in the field of clinical tumour treatment. The emergence of immunotherapy has instilled renewed optimism in patients with TNBC. Tumour immunotherapy aims to enhance the immune response against tumours by either triggering anti‐tumour immunity or reducing the immunosuppressive state of tumours. One promising approach is the use of EVs, which can effectively deliver tumour‐associated antigens to immune cells and exert an immunotherapeutic effect. Furthermore, EVs can be engineerable. Their surface molecules possess significant modification potential and can be loaded with therapeutic cargoes, such as genes, chemotherapeutics, photosensitizers, or immunomodulators. The incorporation of these cargoes into EVs can enhance the targeting ability and therapeutic efficacy of the engineered nanocarriers [93] (Table 2).

**TABLE 2:** The role of EVs in the immunotherapeutic potential in TNBC.

Key ingredients	Type of EV	Cells of origin	Specific mechanism	Ref. No.
Immune checkpoint				
High‐affinity variant of human PD‐1 protein	EV	Knocking out HLA‐I and PD‐L1 in MDA‐MB‐231 cells	Abolishing PD‐L1‐mediated T cell suppression	[94]
PD‐1, OX40 ligand, a monoclonal antibody specific to T cell CD3 and EGFR	Exosome	Expi293F cells	Enhancing the infiltration of CD8+ T cells and alleviating the population of Tregs	[95]
OX40L	Exosome	Macrophages with heightened expression of OX40L	Reprogramming TAMs into M1‐like macrophages and enhancing the activation and proliferation of CD8+ T cells	[96]
Cancer vaccines				
PTX	MP	Breast cancer cells	Suppressing tumour cell proliferation and inducing apoptosis in tumour cells Activating DCs and polarising M1 macrophages	[97]
Human neutrophil elastase and Hiltonol	Exosome	α‐LA‐engineered MDA‐MB‐231 cells	Inducing ICD by activating cDC1s and enhancing CD8+ T cell responses	[98]
Photosensitizer Ce6 and sEV inhibitor GW4869	sEV	Bone mesenchymal stem cells	Inducing ICD suppressing immune cells (Tregs and MDSCs) and stimulating CD8^+^ T cells by inhibiting the secretion of sEVs	[99]
DOX and siSTAT3	EV	4 T1 cells	Enhancing the induction of tumour ICD by DOX and promoting infiltration of immune cells into the TME	[16]
Chimeric antigen receptor (CAR) immunotherap				
No specific	exosome	MSLN‐targeted CAR‐T cells	Inhibiting tumour growth by secreting perforin and granzyme B	[100]
Transferrin receptor‐binding peptides (T7)	Exosome	CAR‐NK cells	Traversing the blood–brain barrier	[101]

### 6.1 Immune Checkpoint

The use of immune checkpoint pathways by tumour cells to evade immune detection and promote their growth is well known in cancer research. There are two types of immune checkpoint pathways, wherein PD‐1/PD‐L1 functions as an inhibitory pathway while OX40 (also known as CD134) /OX40L serves as an activating pathway [96]. To counteract this, researchers have focused on immune checkpoint blockade (ICB) as a potential strategy to enhance the immune response and eliminate tumour cells. Recent studies have shed light on the role of EVs in ICB. For instance, researchers have generated engineered EVs called havPD‐1 EVs. They modify MDA‐MB‐231 cells to knock out human leukocyte antigen class I (HLA‐I) and PD‐L1 expression, while simultaneously overexpressing a high‐affinity variant of human PD‐1 protein (havPD‐1). These engineered havPD‐1 EVs effectively abolish PD‐L1‐mediated T cell suppression and exhibit potent anti‐tumour efficacy in xenograft tumour models [94]. In a similar approach, researchers have engineered multifunctional immunomodulatory exosomes with modified surfaces expressing PD‐1, OX40 ligand (OX40L), a monoclonal antibody specific to T cell CD3, and EGFR. These modified exosomes have demonstrated the ability to induce a strong cellular immune response against EGFR‐positive TNBC tumours. They achieve this by enhancing the infiltration of CD8^+^ T cells and alleviating the population of Tregs [95].

Furthermore, based on the OX40/OX40L pathway, the researchers constructed macrophages with heightened expression of OX40L to acquire exosomes secreted by M1‐like macrophages. These exosomes directly acted on TAMs, reprogramming them into M1‐like macrophages, and also bound to OX40 on T cells to enhance the activation and proliferation of CD8^+^ T cells. This synergistic effect of innate and adaptive immunity enables a potent anti‐tumour response [96].

### 6.2 Cancer Vaccines

Cancer vaccine is based on tumour antigens to deliver antigenic information to DCs, thereby inducing long‐lasting anti‐tumour immunity. A novel strategy has been developed for the treatment of TNBC by combining tumour cell‐derived microparticles loaded with PTX (MP‐PTX) with radiotherapy. This combination therapy not only suppresses tumour cell proliferation and induces apoptosis in tumour cells but also mitigates the immunosuppressive microenvironment at the tumour site by activating DCs and polarising M1 macrophages [97].

The induction of ICD is another crucial aspect in tumour immunotherapy, as it facilitates DCs maturation and augments the infiltration of cytotoxic T lymphocytes to induce anti‐tumour immunogenicity [16]. Researchers have developed an in situ DC vaccine called HELA‐Exos. This vaccine is created by loading inducers of ICD (human neutrophil elastase and Hiltonol, a TLR3 agonist) into exosomes derived from alpha‐lactalbumin (α‐LA)‐engineered MDA‐MB‐231 cells. HELA‐Exos is specifically targeted to the tumour microenvironment and induces ICD by activating conventional type 1 dendritic cells (cDC1s) in TNBC cells, thereby enhancing CD8^+^ T cell responses against tumours [98]. Novel immunomodulatory photosensitive nanovesicles (Ce6‐GW4869/sEVs) are developed for encapsulating the photosensitizer chlorin e6 (Ce6) and sEV inhibitor GW4869 in bone marrow mesenchymal stem cell (BMSC)‐derived sEVs through electroporation. These nanovesicles exhibit TNBC‐targeting ability and can effectively induce ICD in TNBC, thereby reversing the immunosuppressive TME. In addition, GW4869 suppresses the activation and proliferation of immunosuppressive immune cells (Tregs and MDSCs) and stimulates the activation and proliferation of CD8^+^ T cells by inhibiting the secretion of sEVs [99]. A synergistic therapeutic approach is established, integrating gene therapy, chemotherapy, and immunotherapy based on signal transduction and the activation of the transcription factor 3 (STAT3) short interfering RNA (siSTAT3) and DOX functionalized tumour‐derived EVs (siSTAT3‐DOX@TEV). It targets tumour tissues precisely, enhances the induction of tumour ICD by DOX, and promotes the infiltration of immune cells into the TME, thereby effectively triggering an anti‐tumour immune response [16].

### 6.3 Chimeric Antigen Receptor (CAR) Immunotherapy

The application of CAR cell‐derived EVs has demonstrated remarkable advancements in the field of breast cancer treatment. Exosomes derived from mesothelin (MSLN)‐targeted CAR‐T cells have been shown to effectively target MSLN‐positive TNBC cells and inhibit tumour growth by secreting perforin and granzyme B [100]. Additionally, the CAR‐NK cell‐derived exosomes and transferrin receptor‐binding peptides (T7) can form nanoplatforms capable of traversing the blood–brain barrier and selectively exerting anti‐tumour effects on HER2‐positive breast cancer cells in the brain [101].

In conclusion, EV‐carried cargoes remodel the TME and augment immunotherapy sensitivity. Moreover, the inherent advantages of EVs demonstrate significant potential in future TNBC immunotherapy.

## 7 Engineered EVs as Drug Carriers for TNBC Treatment

The excellent designability of EVs, combined with their low immunogenicity, high biocompatibility, anti‐biodegradation property, and targetability, enables their integration with existing therapies. These make them a promising drug delivery system for oncology treatment (Table 3).

**TABLE 3:** Engineered EVs as a drug carrier for TNBC treatment.

Key ingredients	Type of EV	Cells of origin	Ref. No.
Engineered EVs loaded with chemotherapeutic agents			
Poly lactic‐co‐glycolic acid, DOX and peptide targeting the c‐Met	Exosome	Macrophages	[102]
HER2	EV	BT‐474 cells with HER2 overexpression	[103]
EVs loaded with Non‐coding RNAs			
Cationic bovine serum albumin and siS100A4	Exosome	Autologous breast cancer cells	[104]
miR‐335	EV	B cells	[105]
DOX, cholesterol‐modified miRNA 159, disintegrin and metalloproteinase 15	Exosome	Human monocyte‐derived macrophage cells	[106]
cRGD, miR‐588	Exosome	Human mesenchymal stem cells	[106]
miR‐3182	Exosome	Human umbilical cord MSCs	[107]
miRNA‐125b	Exosome	Wharton's Jelly MSCs	[108]
miR‐218 mimics	Exosome	Adipose MSCs	[109]

### 7.1 Engineered EVs Loaded With Chemotherapeutic Agents Mitigate the Adverse Effects

Due to the lack of tissue specificity, conventional chemotherapy exhibits significant systemic toxicity in clinical TNBC patients. EVs‐based drug delivery enhances the accumulation of drugs in targeted cells, thereby improving treatment efficacy and reducing cellular toxicity. Li et al. [102] developed a macrophage‐derived exosomes‐coated Poly lactic‐co‐glycolic acid (PLGA) nanoparticles, loaded with DOX, which were further modified with a peptide to target the mesenchymal‐epithelial transition factor (c‐Met). The engineered exosomes enhanced the cellular uptake efficiency and the antitumor efficacy of DOX, enabling targeted chemotherapy for TNBC cells. In a novel investigation, the researchers successfully achieved targeted delivery of sufficient HER2 to the surface of MDA‐MB‐231 cells by EV‐plasma membrane fusion using HER2^+^ EVs derived from BT‐474 cells with HER2 overexpression, and thus conferring MDA‐MB‐231 cells with targetable receptors for selective delivery of chemotherapeutic agents. Consequently, the combination of HER2 targeting and PTX demonstrated significant efficacy for both in vitro and in vivo [103].

### 7.2 EVs Loaded With Non‐Coding RNAs for Precise Gene Therapy

Non‐coding RNAs, such as small interfering RNAs (siRNAs) and inhibitory miRNAs, have shown potential as therapeutic options for TNBC. However, their vulnerability to degradation presents a challenge [102, 110]. To address this issue, biomimetic nanoparticles, specifically CBSA/siS100A4@Exosome, have been developed. These nanoparticles consist of cationic bovine serum albumin (CBSA) conjugated to a siS100A4 core and an exosome membrane shell, enabling the effective delivery of therapeutic siS100A4 to the lung. Remarkably, this delivery system successfully suppresses postoperative lung metastasis in TNBC [104].

Another strategy involves the utilisation of tumour suppressor miRNA through genetic modification of cells to incorporate miRNA‐based therapeutic drugs and/or targeting ligands into EVs. Plasmid DNA‐induced EVs loaded with tumour suppressor miR‐335 restored the endogenous miR‐335 pool in TNBC cells, leading to the downregulation of its target gene, SRY‐box transcription factor 4 (*SOX4*) to inhibit tumour growth [105]. Gong et al. [106] have developed a novel nanoscale exosome‐based delivery system that specifically targets TNBC cells. This system utilises exosomes enriched with disintegrin and metalloproteinase 15 (A15), a molecule that binds to the surface integrin αvβ3 of TNBC cells. Through this targeted delivery approach, cholesterol‐modified miRNA 159 and DOX are co‐delivered to TNBC cells, effectively enhancing therapeutic efficacy without causing adverse reactions. MSC‐derived EVs possess inherent tumour homing capability and long circulating half‐life [111]. After cRGD modification, MSC‐derived exosomes loaded with miR‐588 can effectively deliver miR‐588 to TNBC cells and remodel the immunosuppressive TME via anchoring CCL5 [111]. Similarly, studies have shown Human umbilical cord MSC‐exosomes loading miR‐3182 [107], Wharton's Jelly MSC‐derived exosomes containing miRNA‐125b [108] and adipose MSC‐derived exosomes carrying miR‐218 mimics [109] can be internalised by TNBC cells, modulating the expression of target genes associated with tumour growth and metastasis.

In conclusion, EVs demonstrate powerful anti‐tumour effects, effective drug delivery capabilities, and reduced immune response in the treatment of TNBC. This allows for improved targeting of tumours and lessened drug toxicity. In comparison to conventional therapy, EV‐based tumour treatment offers a more precise, safe, and efficient approach, providing additional therapeutic options and renewed hope for TNBC patients in clinical settings.

## 8 Anti‐Tumour Effect of TNBC‐Derived EV MiRNAs

The field of microRNAs has been extensively investigated in cancers. The significance of EV‐encapsulated miRNAs in cancer pathogenesis has been extensively emphasised by numerous studies. The expression levels of miRNAs are typically upregulated in TNBC, and they play a pivotal role in orchestrating tumour progression by targeting specific genes. In this study, EV miRNAs have exerted oncogenic functions in TNBC, encompassing the processes of invasion, metastasis, growth, drug resistance, and immunosuppression. Moreover, they also facilitate the crosstalk between cancer cells and specific cells of the TME such as CAFs, TECs, adipocytes, TAMs, and immune cells to modulate and reshape the TME. However, EV miRNAs also exhibit tumour suppressive properties in TNBC. For instance, the downregulation of EV miR‐4488 expression in the serum of TNBC patients has been observed. The overexpression of EV miR‐4488 exhibits potent anti‐angiogenic properties by targeting CX3CL1 in the metastatic niche, effectively suppressing tumour cell colonisation [112]. Additionally, exosomal miR‐134 [113] and exosomal Let‐7 [114] overexpressed in TNBC cells have been found to diminish the invasive and migratory capacities of TNBC cells by modulating the STAT5B‐HSP90 signalling pathway and suppressing c‐MYC expression, respectively. In conclusion, the meticulous and efficacious regulation of miRNA expression in tumours will exert a favourable impact on tumour therapy.

## 9 EVs As Potential Biomarkers for TNBC

**FIGURE 4:**
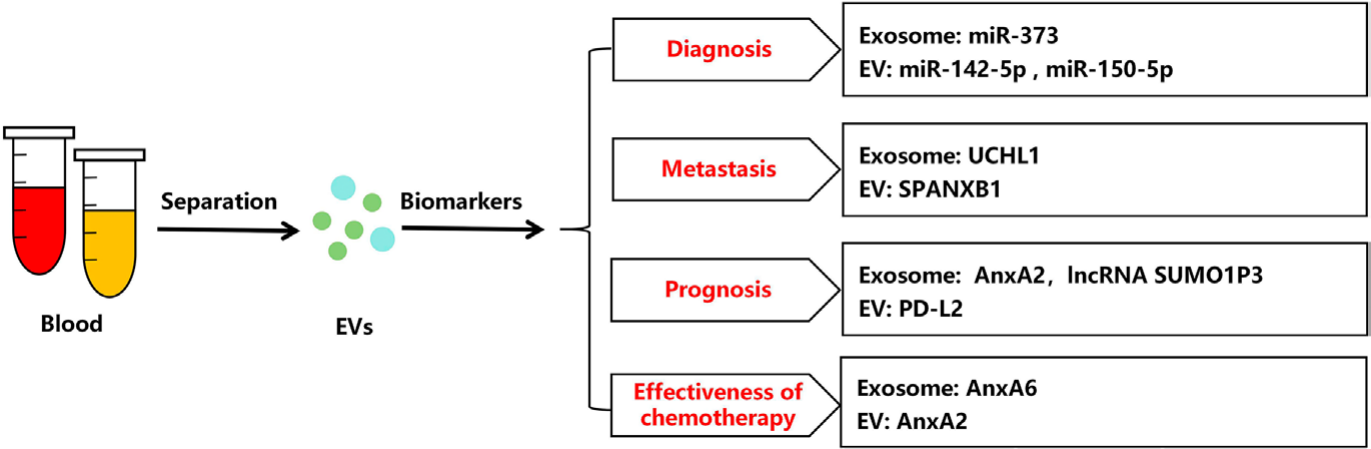
EVs as potential biomarkers for TNBC.

Liquid biopsy is an emerging non‐invasive diagnostic technology that has the potential to revolutionise our understanding of tumours. It achieves this by analysing CTCs, circulating tumour DNA (ctDNA) and EVs in blood or body fluids. These innovative biomarkers pave the way for personalised precision medicine [115]. Compared to other biomarkers like CTCs and ctDNA, EVs offer distinctive advantages. They contain active substances that accurately reflect the biological characteristics of the cells from which they originate. Additionally, EVs are protected by a lipid bilayer structure during blood transport, ensuring the preservation and stability of these active substances. EVs thus serve as superior biomarkers for disease monitoring and prognosis (Figure 4).

### 9.1 Diagnostic Function

EVs derived from TNBC patients displayed distinct profiles of protein and RNA contents compared to those from healthy individuals. Specifically, serum exosomal levels of miR‐373 were significantly higher in TNBC patients compared to other breast cancer subtypes [116]. Moreover, the discriminatory potential between Luminal A and TNBC subtypes was observed through the identification of enriched miRNAs, such as EVs miR‐142‐5p and miR‐150‐5p, in serum EVs from breast cancer patients [117]. These findings indicate that exosomal miR‐373, as well as EVs miR‐142‐5p and miR‐150‐5p, hold promise as potential diagnostic markers for TNBC.

### 9.2 Metastasis and Prognosis

EVs also play a crucial role in predicting the metastasis of cancer and forecasting the prognosis of patients with TNBC. In metastasis, Ubiquitin C‐terminal hydrolase L1 (UCHL1), a protein, promoted the metastasis of TNBC by safeguarding the TGFβ type I receptor and SMAD2 from degradation. Significantly elevated levels of UCHL1 were observed in the blood serum and exosomes of TNBC patients, suggesting that UCHL1‐containing exosomes could potentially serve as a biomarker for early detection of metastasis in tumours [118]. The cancer testis antigen SPANX family member B1 (SPANXB1) was found to be associated with the spontaneous metastasis of TNBC to the lung and liver, and elevated levels of SPANXB1 in the circulating sEVs were significantly linked to advanced clinical stage and histological grade in patients [119].

In prognosis, Chaudhary et al. [120] conducted a comparative study among TNBC patients, ER^+^ breast cancer patients, HER2^+^ breast cancer patients, and controls. They discovered that exosomal annexin A2 (AnxA2) was significantly upregulated in the serum of TNBC patients and correlated with tumour grade and poor survival. Further investigations revealed that high expression of exosomal AnxA2 promoted angiogenesis and enhanced invasiveness in TNBC cells. Furthermore, the serum exosomal long non‐coding RNA small ubiquitin‐like modifier 1 pseudogene 3 (SUMO1P3) emerged as a promising prognostic biomarker in TNBC. It was strongly associated with poor survival, lymphovascular invasion, lymph node metastasis, and histological grade [121]. EVs carrying programmed death ligand 2 (PD‐L2) might serve as a novel biomarker for identifying high‐risk recurrence in early TNBC patients. The expression of PD‐L2 in EVs was observed to be upregulated in TNBC patients, which correlated with a significant decrease in 3‐year progression‐free survival (PFS) and overall survival (OS). Additionally, high expression of EVs PD‐L2 was observed in patients with pre‐chemotherapy Notch1‐positive or CTCs ERBB3‐positive tumours. Subsequent analysis revealed that patients with both CTCs ERBB3 positivity and high pre‐chemotherapy EVs PD‐L2 levels had a shorter PFS [122].

### 9.3 Effectiveness of Chemotherapy

EVs hold great potential as a valuable predictive biomarker for determining the effectiveness of chemotherapy. They can serve as an early indicator to guide medication decisions. In particular, this elevated expression of AnxA2 in sEVs was indicative of a favourable response to neoadjuvant chemotherapy in TNBC patients [123]. Another protein, known as exosomal annexin A6 (AnxA6), has been found to be highly expressed in gemcitabine‐resistant TNBC cells and induces tumour resistance through the inhibition of EGFR ubiquitination and degradation. Clinical studies have revealed baseline levels of exosomal AnxA6 are significantly lower in highly sensitive TNBC patients compared to those with resistance to first‐line gemcitabine‐based chemotherapy [124]. These suggest that the levels of exosomal AnxA6 and AnxA2 in the serum of patients with TNBC might serve as a predictive biomarkers for therapeutic responsiveness to chemotherapy.

EVs carrying cargoes have shown promise as biomarkers of diagnosis and effectiveness of chemotherapy and can influence patients' prognosis, including tumour grade, lymph node metastasis, and distant organ metastasis. Meanwhile, they also provide a basis for identifying highly sensitive and specific markers for detecting metastasis and assessing prognosis.

## 10 The Future Directions and Challenges in EV‐Based Detection and Therapy

The objective of EV research typically varies based on the type of sample, which includes cell culture medium, blood, bodily fluids (urine, cerebrospinal fluid, saliva, nasal secretions, breast milk, etc.), and tissue specimens [6]. For instance, urine may be a great advantage for studying EV markers in genitourinary diseases. Also, the collection and pretreatment of samples have remarkable consequences on the outcomes of EV analysis. There are numerous factors that influence the analysis of EV in blood and body fluids, including the subjects' conditions, the methods and timing of sample collection, the choice of anticoagulant, as well as post‐collection storage procedures [6]. All these factors may result in differences in EV properties, EV populations, and contaminants, ultimately leading to variations in the outcomes.

The enrichment/isolation of EV forms the foundation for downstream analysis and application. Despite the availability of numerous techniques for EV enrichment, isolation, and characterisation, few methods achieve complete purification of EV from other cellular components. Currently, ultracentrifugation is a widely used technique for EV enrichment, which is often combined with ultrafiltration. However, this method has low throughput and may extract non‐target particles with similar physical properties [125]. Commercial kits are available for simpler, high‐yield EV precipitation, but they often introduce many contaminants, such as precipitating agents or protein aggregates [126]. Advanced techniques such as size‐exclusion chromatography (SEC) and immunoaffinity methods increase the isolation of EV purity [127]. Recently, emerging technologies, including microfluidic filtering [7], lipid‐based nanoprobes [128], and dichotomous size‐exclusion chromatography (dSEC) [129], offer distinct advantages over conventional EV isolation methods. These advantages encompass the preservation of EV biological characteristics, purification efficiency, simplified operational procedures, and sample volume requirement. EV still lack universal markers, and existing technologies have yet to achieve an optimal balance among purity, specificity, recovery rate, operational complexity, and cost‐effectiveness.

Once enriched, it is imperative to analyse EV characterisation through a variety of methodologies. Microscopic methods are widely utilised to measure the physical properties of EVs, including vesicle size and distribution, concentration, and morphology. These methods ensure the preservation of EV membrane integrity effectively [7]. Dynamic light scattering (DLS), nanoparticle tracking analysis (NTA), tunable resistive pulse sensing (TRPS), and flow cytometry (FCM) are frequently utilised for the rapid assessment of the physical characteristics of EVs [7]. Protein analyses mainly include western blotting, enzyme‐linked immunosorbent assay (ELISA), mass spectrometry, and FCM. For the analysis of nucleic acids, PCR‐based methodologies, such as droplet digital PCR (ddPCR) and next generation sequencing (NGS), are essential. Also, immunoaffinity capture technology encompasses both enrichment and characterisation capabilities. However, EV subpopulations exhibit heterogeneity, and those negative targeting molecules are excluded [125]. To characterise the heterogeneity of EVs, a number of single EV analysis methods have been developed, including nanoscale flow cytometry [130], droplet microfluidic technology [131], squeezable methacrylated hyaluronic acid hydrogel microparticles (MHPs) [132], and super‐resolution microscopy. In summary, each of these techniques possesses distinct advantages and limitations. By leveraging the complementary strengths of multiple methodologies, it is possible to obtain accurate and comprehensive characterisation data for EVs.

In light of the aforementioned factors, there exists significant heterogeneity in EV research, which compromises the interpretation and reliability of findings. The lack of standardised experimental protocols, along with the absence of reference materials and controls, restricts result reproducibility and impedes interlaboratory comparisons and sharing of results [6]. Consequently, it is imperative to establish a standardised and normative quality control system for EVs in order to enhance the repeatability and reliability of test results. With the development in single EV analysis, more advanced resolution, greater multi‐target analysis capability, and higher‐throughput imaging techniques will substantially facilitate the characterisation of single EVs and provide comprehensive insights into EV composition, enabling more precise delineation of EV subpopulations. Still, there remains a paucity of effective methodologies for the classification and enrichment of EVs. It is crucial to develop efficient, high‐throughput, and multiparametric EV extraction platforms [133]. EVs as novel biomarkers in liquid biopsy require validation through multicenter clinical trials enrolling a substantial number of patients and corresponding controls. With intensive translational research, new EV biomarkers with approved clinical applications and new commercial kits will be expected to be available in the near future. These innovations should be designed for a large volume of samples, affordable instrumentation, simple operation, rapid detection, and automation, and they should also specify the performance characteristics, such as sample type, accuracy, repeatability, and linear range [125].

Recently, novel EV‐based tumour therapies are advancing at a rapid pace. However, the research is still in its preliminary stages and presents several challenges in terms of treatment. First, the heterogeneity of EVs in terms of size and contents presents challenges for their purification and identification, necessitating further studies on capture and analysis techniques. Second, the safety of EVs cannot be determined due to their possession of parental cell properties and the complex and diverse contents. For instance, in the case of tumour‐derived EVs, it is necessary to rule out any carcinogenic effects. To address this issue, a thorough analysis and modification of EVs are imperative for future applications [134]. Thirdly, future discussions should focus on addressing issues related to low yield, insufficient payload, and the validation of targetability and therapeutic efficacy in EV‐based therapies [135].

## 11 Conclusion

TNBC is considered to be the most aggressive subtype of breast cancer that exhibits resistance to conventional treatment regimens. Increasing evidence has indicated EVs exert a significant influence on tumour progression by remodelling the TME and may also become a potential biomarker for early diagnosis, progression, and chemotherapy efficacy in TNBC due to their distinctive capacity for delivering diverse cargoes. The immunomodulatory effect of EVs in TNBC has demonstrated their potential as a promising immunotherapy for combating TNBC. Moreover, engineered EVs have exhibited significant therapeutic efficacy in the treatment of TNBC. Although significant progress has been made in elucidating the pathogenesis of TNBC tumours and exploring the clinical application and treatment potential of EVs, it is important to acknowledge that research in this field is still at an early stage and remains largely unexplored. Therefore, A comprehensive and meaningful study needs to fully comprehend the clinical and therapeutic value of EVs. Moreover, integrating EV‐based advanced therapy with other anti‐tumour modalities might offer patients a wider range of effective treatment options. In conclusion, despite encountering significant challenges in EV‐based therapy against TNBC, the application of EVs will seamlessly integrate into routine clinical practice, with the accumulation of more robust clinical evidence and the establishment of standardised methods for EVs, along with advancements in multi‐omics technology.

## Author Contributions

**Xue Wan:** conceptualization (equal), investigation (equal), visualization (equal), writing – original draft (equal). **Liqi Yang:** investigation (equal), resources (equal), validation (equal), visualization (equal), writing – original draft (equal). **Linjun Wu:** conceptualization (equal), funding acquisition (equal), resources (equal), validation (equal), visualization (equal). **Jiandong Lei:** conceptualization (equal), funding acquisition (equal), investigation (equal), supervision (equal), visualization (equal). **Jintao Li:** conceptualization (equal), resources (equal), supervision (equal), writing – review and editing (equal).

## Conflicts of Interest

The authors declare no conflicts of interest.

## Data Availability

The data that support the findings of this study are available from the corresponding author upon reasonable request.
